# Evaluation of a financial incentive intervention on malaria prevalence among the residents in Lake Victoria basin, Kenya: study protocol for a cluster-randomized controlled trial

**DOI:** 10.1186/s13063-024-07991-4

**Published:** 2024-03-04

**Authors:** Tomoya Matsumoto, Masaru Nagashima, Wataru Kagaya, James Kongere, Jesse Gitaka, Akira Kaneko

**Affiliations:** 1 Department of Economics, Faculty of Commerce, Otaru University of Commerce, Hokkaido, Japan; 2 Institute of Developing Economies Japan External Trade Organization (IDE-JETRO), Chiba, Japan; 3 Department of Protozoology, Institute of Tropical Medicine (NEKKEN), Nagasaki University, Nagasaki, Japan; 4 Department of Ecoepidemiology, Institute of Tropical Medicine (NEKKEN), Nagasaki University, Nagasaki, Japan; 5 Department of Virology and Parasitology, Graduate School of Medicine/Osaka International Research Center for Infectious Diseases, Osaka Metropolitan University, Osaka, Japan; 6 Directorate of Research and Innovation, Mount Kenya University, Thika, Kenya; 7 Department of Microbiology, Tumor and Cell Biology, Karolinska Institutet, Stockholm, Sweden

**Keywords:** Malaria, Malaria education, Conditional cash transfer, Lottery incentive scheme, Kenya, Cluster-randomized controlled trial

## Abstract

**Background:**

In the Lake Victoria basin of western Kenya, malaria remains highly endemic despite high coverage of interventions such as mass distribution of long-lasting insecticidal nets (LLIN), indoor residual spraying (IRS) programs, and improvement of availability and accessibility of rapid diagnostic tests (RDT) and artemisinin-based combination therapy (ACT) at community healthcare facilities. We hypothesize that one major cause of the residual transmission is the lack of motivation among residents for malaria prevention and early treatment.

**Methods:**

This study will aim to develop a demand-side policy tool to encourage local residents’ active malaria prevention and early treatment-seeking behaviors. We examine the causal impact of a financial incentive intervention complemented with malaria education to residents in malaria-prone areas. A cluster-randomized controlled trial is designed to assess the effect of the financial incentive intervention on reducing malaria prevalence in residents of Suba South in Homa Bay County, Kenya. The intervention includes two components. The first component is the introduction of a financial incentive scheme tied to negative RDT results for malaria infection among the target population. This study is an attempt to promote behavioral changes in the residents by providing them with monetary incentives. The project has two different forms of incentive schemes. One is a conditional cash transfer (CCT) that offers a small reward (200 Ksh) for non-infected subjects during the follow-up survey, and the other is a lottery incentive scheme (LIS) that gives a lottery with a 10% chance of winning a large reward (2000 Ksh) instead of the small reward. The second component is a knowledge enhancement with animated tablet-based malaria educational material (EDU) developed by the research team. It complements the incentive scheme by providing the appropriate knowledge to the residents for malaria elimination. We evaluate the intervention’s impact on the residents’ malaria prevalence using a cluster-randomized control trial.

**Discussion:**

A policy tool to encourage active malaria prevention and early treatment to residents in Suba South, examined in this trial, may benefit other malaria-endemic counties and be incorporated as part of Kenya’s national malaria elimination strategy.

**Trial registration:**

UMIN000047728. Registered on 29th July 2022.

## Introduction

### Background and rationale {6a}

Since around 2000, the United Nations/Millennium Development Goals (MDGs) and the establishment of the Global Fund have increased funding for global malaria control. There have been significant improvements in the supply side of healthcare services for malaria, including the distribution of long-lasting insecticidal nets (LLINs), the implementation of indoor insecticide spraying (IRS), the use of rapid diagnostic tests (RDT) at community health facilities, and the use of Artemisinin-based Combination Therapy (ACT) in community health facilities. The number of malaria cases and deaths worldwide has decreased significantly to the point where malaria elimination by 2030 was one of the 2015 United Nations Sustainable Development Goals (SDGs) [1, 2]. However, while malaria prevalence in Southeast Asian countries has been shrinking significantly, tropical Africa continues to experience high malaria prevalence [3]. Moreover, the number of deaths and patients worldwide has leveled off, along with the headwind of funding for disease control since around 2015. In addition, many resources have been diverted to the fight against new coronavirus infections, raising the specter of another malaria pandemic. The WHO reports that the pandemic of new coronavirus infections has put the operation of basic health systems, including malaria prevention, diagnosis, and treatment, at risk. The result has been a measured excess of deaths from malaria of about 47,000 people [3]. How to steer and accelerate the fight against malaria in the direction of global elimination once again is one of the most significant global health challenges we face in a post-corona era, and malaria control in tropical Africa is a clear focus of this effort.

This project targets residents of Suba South Sub-County, Homa Bay County, along Lake Victoria in western Kenya, where malaria prevalence remains high. According to the results of a rapid malaria diagnostic test in the census (7060 subjects) conducted by our team (December 2022–February 2023), the positive rate was 16.7%, and even higher for children under 15 years old (24.4%). In the area with the highest infection rate, 69 out of 102 (68%) were positive. Despite the fact that malaria control efforts by national and international aid agencies have been returning to pre-corona pandemic levels, the prevalence rate remains very high in this region.

One of the important factors for the stagnation of the downward trend of malaria infection exists on the demand side, that is, people who use healthcare services for malaria control. The inappropriate and suboptimal uses of LLINs are commonly observed among the residents in the region [4–6]. Thus, it is clear that the top-down intervention, e.g., free mosquito net distribution or IRS program, alone has a limitation in eliminating malaria from the areas. It is necessary to consider measures to encourage preventive actions on the demand side of healthcare. Existing studies suggest that people tend to underinvest in preventive healthcare and spend more on treatment costs of illness [7]. Moreover, people tend to make less effort for preventive healthcare for communicable diseases such as malaria than socially optimal level due to inconsideration of the health of others by ignoring the fact that their own prevention efforts reduce the infection risk of their neighbors [8–10]. We hypothesize that the stagnation of the malaria reduction trend is caused by the fact that residents in malaria-prone areas underestimate the value of preventive healthcare due to the lack of knowledge of benefits from the care and also oversight of direct and indirect costs of illness. In order to address the problem, it is crucial that the residents themselves understand the benefits of malaria prevention and maintain their appropriate use of preventive measures for a long. This project aims to encourage residents to use appropriate malaria prevention methods and seek early treatment continuously through a financial incentive scheme in which a monetary reward is given to those with negative testing results of malaria infection, complemented with malaria education through a tablet-based animated material that we develop.

A financial incentive intervention combined with malaria knowledge education we plan in this study can motivate residents to make further efforts for malaria prevention. Here, we designed a cluster-randomized controlled trial to evaluate its effect on fostering behaviors of malaria prevention and early treatment and reducing malaria prevalence. Thus, we will monitor the behaviors and prevalence of the target residents. The most vulnerable population to malaria is small children, whereas adults are also an important population in malaria transmission since they can act as a reservoir of transmission as asymptomatic infections. At the same time, adults are the ones who decide how they handle malaria prevention and treatments in the family. Thus, the proposed study includes monitoring two target populations: children (zero to 15 years old) and all age groups.

### Objectives {7}

The primary study objective is to evaluate the causal impact of the financial incentive intervention on malaria prevalence in children aged zero to 15 and all age groups during a 6-month follow-up period. The secondary objectives during a 6-month follow-up period are.

1. To measure the impact of the financial incentive intervention on malaria preventive behaviors, especially bed net usage after the 6 months of the intervention,
2. To measure the impact on malaria knowledge of target individuals and
3. To measure the spillover effects from those exposed to the intervention to their geographic neighbors and those who are socially connected.

### Trial design {8}

The study hires a two-stage randomized controlled trial with 92 clusters with three arms for impact evaluation. In this study, we have two different forms of financial incentive schemes, CCT and LIS, combined with malaria education (EDU), consisting of two intervention arms, while the third arm is for control. We randomly assign these three arms at the cluster level at the first stage.

Clusters are defined as a set of 20 adjacent households based on the household location information obtained in the household census conducted in coastal communities of the Suba South Sub-County from May to July 2021 and include 5968 households. Although there are numerous ways to define 92 clusters with 20 adjacent households from 5968 households in the region, we randomly select a combination of 92 clusters in a way that none of the cluster-comprising households belong to more than one cluster.

**Fig. 1:**
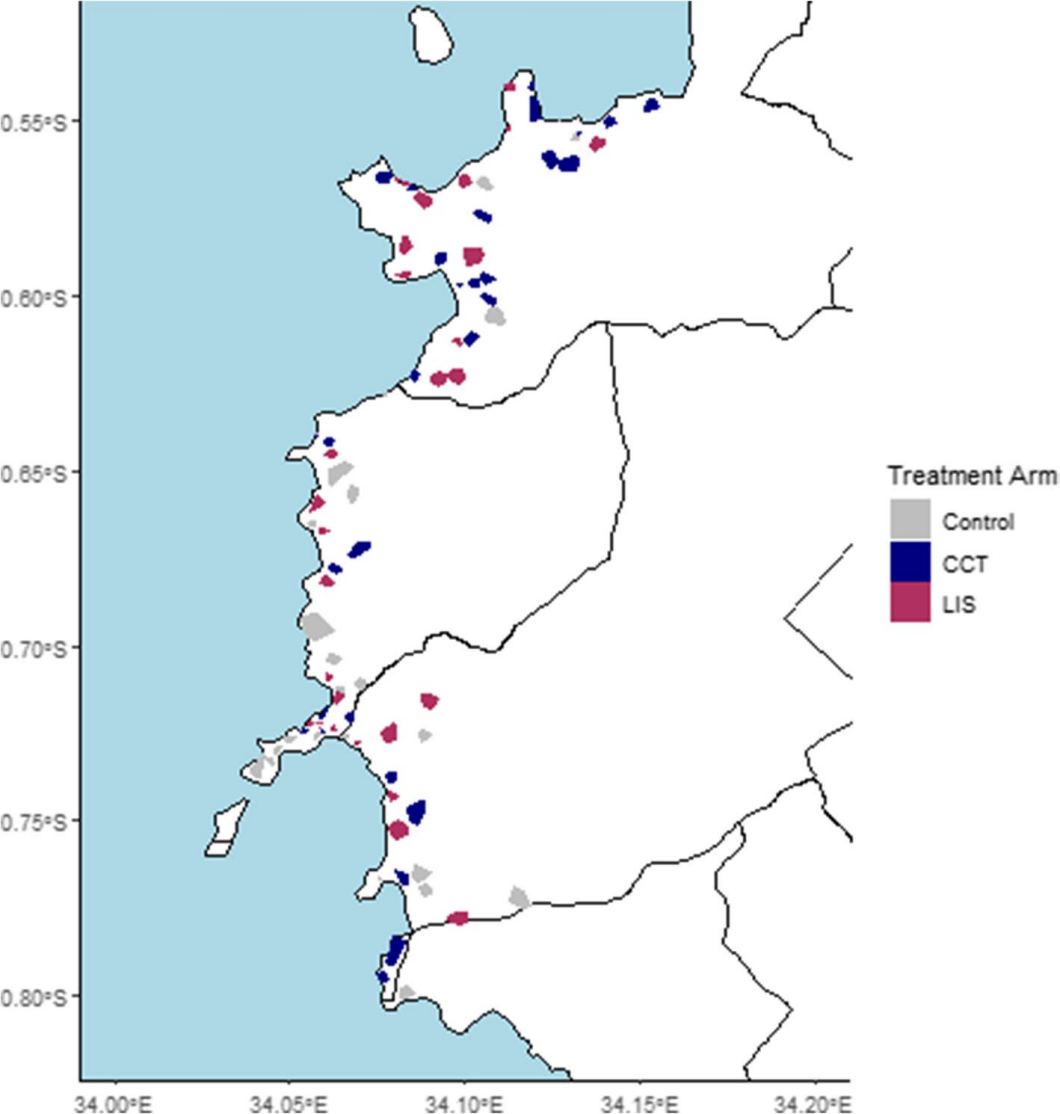
Map of Suba South showing the locations and the types of arms of the 92 trial clusters

Once we define the clusters, they are stratified by the average malaria prevalence and the number of children under 15 years of age in the clusters based on the baseline survey, and one of the three arms is assigned to each of the clusters per stratum based on random numbers generated by a priori on the computer. Figure 1 shows the locations of the clusters on the map and arm types by colors.

The randomization at the second stage is done at the household level in the two intervention arms, CCT and LIS, to determine which households will receive the treatment within an intervention cluster. It is designed to measure spillover effects within an intervention cluster from the households with treatment to those without treatment. The random assignment at the household level within an intervention cluster is made by asking a household representative to draw a scratch card during a site visit by the survey team for the intervention. The card has a single number from 1 to 10 printed under a seal. If an even number appears on the card drawn, the household will receive the treatment, meaning that (1) the household receives the malaria education (EDU) at the site visit and (2) its household members become eligible for an incentive scheme (CCT or LIS depending on the arm of the cluster). The household will not receive the treatment otherwise.

A power analysis was conducted after taking into account the size of the expected intervention effect, the number of clusters, and the number of households and individuals following a recent examining regression models for a random saturation experiment [11], which fits our two-stage randomization setting. The two intervention arms, CCT and LIS, are randomly assigned to 32 clusters, respectively, while the control arm is assigned to 28 clusters. The cluster-level randomization is supplemented by intra-cluster random assignment at the household level, and the overall structure of the experiment forms a two-stage randomized control trial.

## Methods: participants, interventions, and outcomes

### Study setting {9}

The study will be conducted in Suba South Sub-County, Homa Bay County, Kenya. According to the most recent national census in 2019, Suba South Sub-County has a land area of 634.1 km^2^ and a population of 122,383 in 27,635 households [12]. Most of the population belongs to the Luo ethnic group, except some residents belonging to Suba ethnic group. The primary occupations of people are fishing in the lake and farming. Most of the household possesses more than two house structures. The main dwelling units are typically made of mud walls with metal sheet roofs, although units with walls made of iron sheets, concrete, or stones are also common [12].

In general, the Lake Victoria region has two rainy periods annually: the long rainy season from March to June and the short rainy season from August to October, though irregular patterns have been observed in the last few years. Malaria incidence peaks 1 to 2 months after the rainy season. The major vectors are *Anopheles gambiae* s.s., *An. arabiensis*, and *An. funestus* [13].

Suba South Sub-County has 30 public health facilities (2 county hospitals, 8 health centers, and 20 dispensaries). The area is divided into health units, each of which consists of 50 to 100 households. The health status of residents in each health unit is monitored by a community health volunteer (CHV). Under a Homa Bay County Government mandate, CHVs are to test suspected malaria cases with an RDT and administer the ACT to treat confirmed positive cases. LLINs are distributed for free by the government periodically, and the next distribution is planned for early 2021.

### Eligibility criteria {10}

In this study, we plan to have a baseline household survey with a blood sampling of the household members, a financial incentive intervention, and a follow-up household survey with a blood sampling of the household members.

The inclusion criteria for the baseline survey are (1) the households of at least one permanent resident aged 18 years or older at the time of the census in the coastal communities in Suba South Sub-County, where the census was conducted and (2) informed consent provided by at least one adult in the household. The inclusion criteria for the baseline blood sampling are (1) members residing in the households that participate in the baseline survey and (2) informed consent provided by themselves or the parent or legal guardian. The exclusion criterion is having severe chronic illnesses. The inclusion criteria for the financial incentive intervention are (1) members of the households that participate in the baseline survey and agree to participate in the follow-up survey and (2) informed consent provided by at least an adult member. The inclusion criteria for the follow-up survey are (1) households in which at least an adult member provides informed consent both in the baseline survey and the financial incentive intervention and (2) informed consent provided by at least one adult in the household. The inclusion criteria for the follow-up blood sampling are (1) members residing in the households that participated in the baseline survey and (2) informed consent provided by themselves or the parent or legal guardian. The exclusion criterion is having severe chronic illnesses.

Table 1 summarizes these criteria.

**Table 1:** Inclusion and exclusion criteria for the intervention and prospective cohort survey

Inclusion criteria	Exclusion criteria
**Baseline (survey)**	
At least one permanent resident aged 18 years or older in the household	
Informed consent provided by at least one adult in the household	
**Baseline (blood sampling)**	
Members residing in the households participated in the baseline survey	Severe chronic illnesses
Informed consent provided by the parent or guardian before each survey	
**Financial incentive intervention**	
Household members residing in the households participated in the baseline survey	
Informed consent provided by at least one adult in the household	
**Follow-up (survey)**	
Households participated in the baseline survey	
Informed consent provided by at least one adult in the household	
**Follow-up (blood sampling)**	
Household members residing in the households participated in the baseline survey	Severe chronic illnesses
Informed consent provided by the parent or guardian before each survey	

### Who will take informed consent? {26a}

Written informed consent will be obtained by study team members fluent in the local languages (Luo), Swahili, and English and who fully understand the study protocol. After eligibility is confirmed, study team members will present to the potential participants a document containing all relevant information about the study in Luo and English. If the participant cannot read, study information will be conveyed verbally by a study team. The potential participants will have opportunities to ask any questions. Agreement to participate is sought only after the participant indicates an appropriate understanding of the study.

### Additional consent provisions for collection and use of participant data and biological specimens {26b}

The study information document for the financial incentive intervention contains the study overview. In addition, the documents for baseline and follow-up surveys and blood samplings contain details on collecting, storing, and using personal data and biological specimens during the study.

## Interventions

### Explanation for the choice of comparators {6b}

In Kenya, LLIN is the most widely used malaria preventive measure. The Division of National Malaria Programme coordinates free LLIN distribution, and the county governments deliver LLINs to residents in all endemic counties every 3 years. The primary purpose of this trial is to evaluate two forms of financial incentive intervention combined with malaria education and their spillover effects. Thus, all the target households will have no restrictions on their behaviors and actions, such as the use of malaria prevention measures and treatment-seeking behaviors. Therefore, although households in the control arm and the untreated households in the intervention arms will have neither incentive nor malaria education, they will be expected to take the current best practices. As for the planning stage, there is no plan for new LLIN distribution during the study period.

### Intervention description {11a}

After the baseline survey and blood sampling, the survey team will have site visits to the households in all the target 92 clusters for the intervention. In the control clusters, the survey team will explain the follow-up survey that will be held in 6 months, confirm their continuous participation in the study, and conduct a short interview in the local language, Luo. In the intervention clusters, in addition to the explanation of the follow-up survey and the confirmation of the participation, the survey team will inform that the intervention would be piloted to the household, that the eligibility for the intervention would depend on a number of a scratch card to be drawn by a household representative, that an even number will make the household eligible for the intervention, and that an odd number will make the household ineligible. The survey team will explain the reward scheme of the CCT in the CCT clusters and that of the LIS in the LIS clusters, respectively, and the timing of the follow-up survey (in 6 months). If the household representative, typically the household head or spouse, understands the incentive scheme correctly, he/she will be asked to draw a card from an envelope containing ten cards. If he/she does not understand, the survey team will explain the rule again before he/she draws a card. If an even number is drawn, he/she will be informed that the household receives the treatment, i.e., its members become eligible for the reward scheme and asked to watch the malaria education material on a tablet device that the survey team will bring, and then proceed to a short interview. Otherwise, he/she will be informed that the household does not receive the treatment, and the survey team will conduct a short interview only. Appendix has the instruction script for enumerators in the intervention to be used.

### Criteria for discontinuing or modifying allocated interventions {11b}

Since the financial incentive intervention is a promise to the eligible household members that the project will give rewards conditioning on their malaria RDT negative status in the follow-up blood sampling conducted in 6 months, it will not cause any health risks to the participants. Thus, the intervention will be discontinued only when the participants request their disinvolvement from the study or the participants migrate out of the coastal communities of Suba South Sub-County, where the census was conducted. We do not allow any crossover from the control arm to the intervention arm during the follow-up period. Those who migrate between the arms or emigrate from the study areas will be dropped from the study follow-up.

### Strategies to improve adherence to interventions {11c}

Adherence to the intervention in this study is defined as being members of eligible households of the financial incentive intervention during the period between the intervention and the follow-up. Adherence is monitored by the study team at the blood sampling visit at the follow-up to collect the blood samples.

### Relevant concomitant care permitted or prohibited during the trial {11d}

There is no specific concomitant care prohibited during the trial. All participants in all the arms will continue to receive and use free LLIN and have access to standard medical care, including malaria testing by RDT and treatment with ACT provided by the private and public service sectors.

### Provisions for post-trial care {30}

All participants will be under the normal healthcare system in the study setting. No perceived health risks for the intended population are expected with the intervention. Our plan of continuous cross-sectional malaria surveillance after the study period allows us to monitor further parasite transmission in the population.

### Outcomes {12}

The primary outcome of the study is malaria prevalence by PCR in children (0 to 15 years old) 6 months after the financial incentive intervention. The secondary outcomes are (i) malaria prevalence by PCR all the age groups for 6 months post-intervention; (ii) microscopic malaria prevalence in children and all the age groups at 6 months post-intervention; (iii) the proportion of the participants who sleep under the bed net at 6 months post-intervention; and (iv) malaria knowledge among the adults, which will be tested using a malaria quiz based on the malaria education contents.

### Participant timeline {13}

**Fig. 2:**
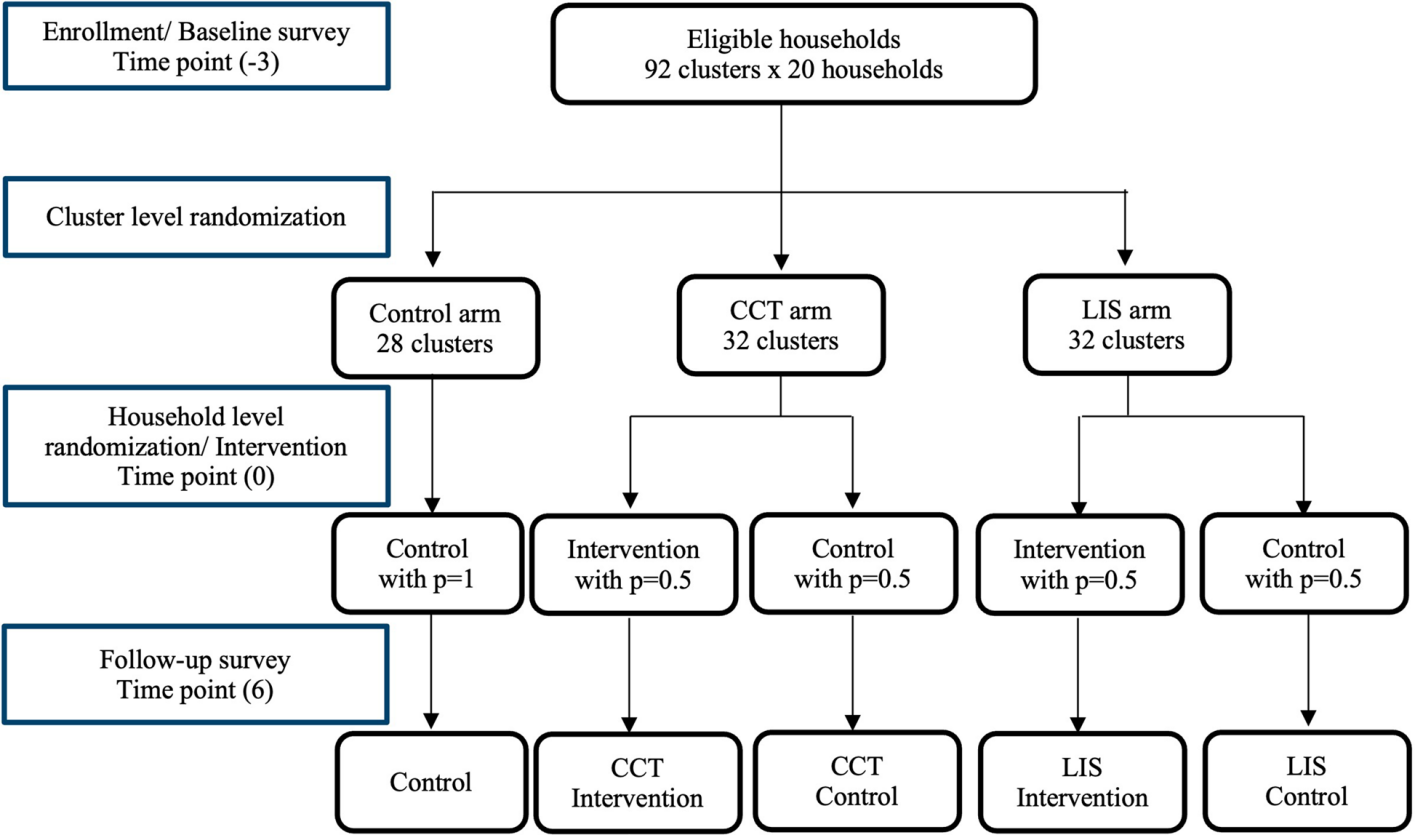
The study flowchart and sampling timeline

The study flowchart is presented in Fig. 2, and the study timeline is presented in Table 2.

**Table 2:**
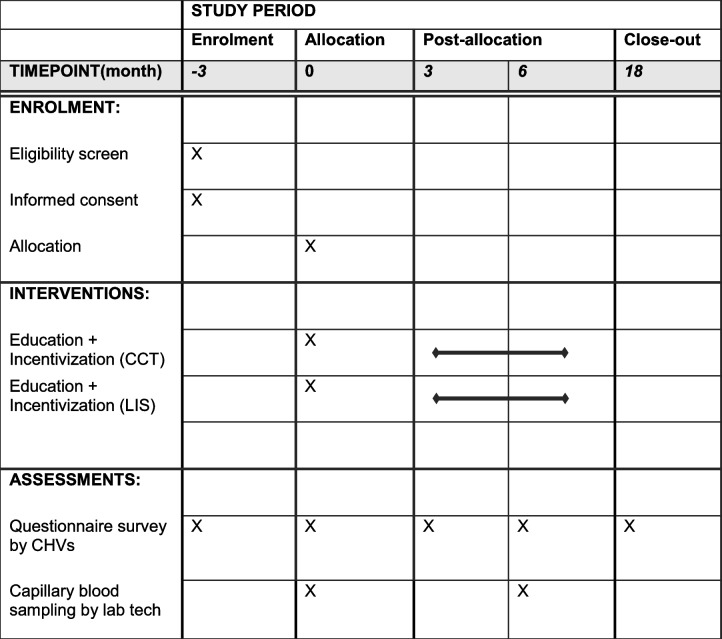
Study timeline

### Sample size {14}

The malaria RDT positivity among residents in our target area is approximately 30%, based on an independent survey conducted at the same time in our study area. Our census survey suggested an approximate average household size of 5. We aim to detect the expected effect of 30% RDT positivity reduction (to 21%) relative to control both in the direct and spillover effects, with a power of 0.8, a two-sided type-I error of 5%, a cluster size of 20 households, and an intra-cluster correlation coefficient of 0.05. We rely on the optimal saturation [11] and find the required number of clusters is 28 for the control arm and 32 for the treatment arm, where ten households in a treatment cluster end up receiving the treatment, and the remaining ten households are left untreated. We have two intervention arms, each of which to compare to the control arm. Therefore, our total intervention sample consists of 9200 individual subjects from 1840 households grouped into 92 clusters.

### Recruitment {15}

#### Community sensitization

We first sought approval from the Homa Bay County Government Ministry of Health and, based on their advice, hosted meetings with CHVs, village chiefs, and Public Health Officers in Suba South Sub-County, and other key stakeholders from the county to explain the purpose, scope, objectives, methods, timeline, and potential significance of our study. CHVs and village leaders were asked to disseminate study information to and answer questions from community members. Feedback from CHVs and village leaders was sought, and regular meetings were held among CHVs, village leaders, and the study team to devise and refine approaches to engage communities. Finally, broad-level community consent to participate in this experimental intervention was sought through CHVs and village chiefs.

#### Community census

After community sensitization and affirmation of agreement to participate provided by village chiefs, a census is conducted by CHVs and experienced local research assistants to enumerate and record demographic information from all households in the health units that are receptive to our study. The following information is collected from each household: (1) the number of residents; (2) the age, gender, and occupation of each resident; (3) the number, type, size, and functions of house structures; (4) current LLIN ownership and usage; and (5) GPS coordinates of the household. Written informed consent to participate in this intervention project will be sought from the head of each household during census house visits.

## Assignment of interventions: allocation

### Sequence generation {16a}

Random numbers that follow uniform distribution between 0 and 1 are generated using R, the statistical software, and assigned to each cluster of 20 compounds. The seed of the quasi-random number is set to a 8-digit integer of “*yyyymmdd*” format, where “*yyyy*” is the year, “*mm*” is the month, and “*dd*” is the day of the date of conducting randomization. Therefore, the seed would be 20,210,930 if the randomization was to be done on the 30th of September, 2021. For this procedure, R built-in functions set.seed() and runif() are to be used.

### Concealment mechanism {16b}

Each cluster is assigned the computer-generated quasi-random number generated in the way described above after the stratification of two baseline indicators: the number of children aged 0 to 15 and their malaria prevalence. The stratification creates four (2 × 2) strata. Then, clusters are arranged in ascending order of their assigned random numbers by stratum. In each stratum, seven clusters with the smallest random numbers are assigned to the control arm, 8 clusters with the next smallest numbers are assigned to the CCT arm, and 8 clusters with the largest random numbers are assigned to the LIS arm. There will be 28 control, 32 CCT, and 32 LIS clusters in total.

### Implementation {16c}

For the arm assignment to clusters, the allocation sequence and random assignment are generated by a volunteer who has no knowledge about the study. Local study assistants will perform participant enrollment.

## Assignment of interventions: blinding

### Who will be blinded {17a}

Firstly, the arm assignment to clusters will not be disclosed to the trial participants. Thus, the trial participants do not know what arm options exist other than the arm assigned to the cluster to which the household belongs. However, there is a possibility that the information on the arm assignments can be known by the trial participants through social interactions across clusters. We try to collect such information flow across clusters in surveys.

Secondly, the treatment assignment within the intervention arms (CCT and LIS arms) will be done at the household level by using a randomly drawn card by the household representatives themselves. Therefore, the household representatives in the intervention arms know if their household belongs to one of the intervention arms and their treatment status.

Thirdly, the study team members who participate in field activities cannot be blinded since each covers several clusters with different arm assignments and implements the household-level randomization for the treatment in the intervention arms. However, laboratory- and office-based personnel (e.g., microscopists and laboratory technicians) will be blinded to the identity and intervention status of the trial participants since all biological specimens will be identified by a unique numeric study identifier, and personal information will be removed before analyses.

### Procedure for unblinding if needed {17b}

This is a socioeconomic intervention providing financial incentives and malaria education, and hence, no physical damages are expected to occur to the participants with the treatment. Therefore, there is no circumstance that they need to be unblinded.

## Data collection and management

### Plans for assessment and collection of outcomes {18a}

#### Baseline and follow-up surveys

Malaria prevalence will be estimated using cross-sectional malariometric surveys. These surveys will be conducted at baseline (before the intervention) and 6 months post-intervention. Malaria status will be determined using three methods: RDT, microscopy, and PCR. First, a finger prick blood sample will be collected for on-site diagnosis by Paracheck-Pf® RDT (Orchid Biomedical Systems, India). Survey participants with positive test results will receive a treatment course of artemether-lumefantrine with dosing instructions as per the guidelines from the Ministry of Health in Kenya. Blood smears will be prepared on-site and transported to the main laboratory in Homa Bay, where thin smears are fixed with methanol. All smears are stained with 3% Giemsa solution for 30 min and then examined by experienced microscopists. Two blood samples (70 µl each) will be collected with a 75-mm EDTA-coated micro-hematocrit capillary tube (Marienfeld, Lauda-Königshofen, Germany) and spotted on Whatman ET31 Chr filter paper (Whatman International. Maidstone, UK). The blood spots will be allowed to dry at ambient temperature and stored in individual zipped plastic bags at − 20℃. The dried blood spots (DBS) will be used for DNA extraction and determination of malaria status by PCR [14].

Malaria incidence will be assessed at the baseline and follow-up after 6 months post-intervention. CHVs will visit the households of the participants. A structured questionnaire created using the CSPro application and loaded on an Android-based tablet computer will be used to collect any history of fever, malaria episode, or visit to the local health facilities in the previous 3 months [15].

Training sessions will be held for CHVs to familiarize themselves with the questionnaire’s content and the CSPro application’s use to record the responses. Built-in validation and completion checks will ensure data quality and completeness, respectively. To avoid duplication, all microscope slides, filter papers, and sample tubes will be pre-labeled with auto-generated serial numbers. CHVs and certified medical laboratory staff will be prompted by the CSPro application before blood sampling to confirm the identity and serial number of the cohort participant. The completeness of blood sampling will be confirmed twice after the sampling step in the field and at the sample storage step in the laboratory.

#### Acceptability of the financial incentive schemes

Focus group discussions (FGDs), a structured questionnaire, and in-depth interviews will be used to assess the acceptance of the financial incentive schemes. At the end of the 6-month follow-up, a representative of all the target households will be subjected to the structured questionnaire, and a part of them will be invited to in-depth interviews based on their responses to the questionnaires.

### Plans to promote participant retention and complete follow-up {18b}

In this survey, CHVs will make an appointment with participants and confirm their available date and time at least a week before each household visit. The participants will receive a small remuneration (i.e., sugar, rice, beans, cooking oil, or soap) after the follow-up survey. CHVs will be instructed to relay to the research team any issue raised by cohort participants, and discussions will be held to resolve issues that cannot be immediately addressed. The research team will periodically accompany the CHVs in their home visits to reinforce to cohort participants the importance of the study.

### Data management {19}

Study data will be collected on Android-based tablet computers using the CSPro application to promote data quality and security. Data validation, such as range checks and completeness checks, will be enabled in all survey instruments. For the baseline and follow-up surveys, data will be uploaded to the CSPro server at the conclusion of each survey day. After the data manager confirms the data quality on the server, data stored locally on the tablet computers will be deleted before the next survey to avoid potential overwriting of existing data. Survey data will be uploaded to the CSpro server at least once a week for the longitudinal cohorts. Each participant is given a unique identifier, and each visit is preprogrammed as a defined event in the CSpro data collection instrument to facilitate data entry. The surveys will be conducted by CHVs familiar with the participants and will be prompted to confirm the identity of the participants before data entry. In addition, the data manager will confirm the data quality on the server once a week. Access to survey data will be limited to data analysts and the data manager in the research team. In addition, personally identifiable information will be removed before data analyses.

### Confidentiality {27}

To maintain confidentiality, each participant in this study is assigned a unique identifier. The data collected will be labeled using the unique identifier and stored separately from the key linking personal information (name, date of birth, GPS, and phone number). The data will be kept on a secure server that is only accessible to research staff. Publications will contain only aggregated data, and no personal information will be included.

### Plans for collection, laboratory evaluation and storage of biological specimens for genetic or molecular analysis in this trial/future use {33}

Blood samples will be collected to examine malaria infections by multiple methods, immunity against malaria parasites and mosquito saliva, and malaria parasite genomics. No human genetic studies are planned in this study. However, any biological specimens remaining after analyses described in this study will be stored indefinitely for future studies unless the participants opt out during the informed consent process. Contact information of the study team is provided in the consent form to study participants, who can remove themselves from this study and any future studies that may use their blood samples at any time without penalty or prejudice.

## Statistical methods

### Statistical methods for primary and secondary outcomes {20a}

The intention-to-treat (ITT) analysis is the primary analysis approach for both the primary and secondary outcomes and the per-protocol analysis will be included as a supplementary analysis.

The primary outcome of the study is malaria prevalence by PCR of children aged 0 to 15 6 months after the financial incentive intervention. Based on the PCR examination results, we apply a regression model for two-stage randomized control trial settings, proposed by a study [11], which allows us to identify an unbiased estimate of the ITT and also the spillover effects on treated and untreated households within a cluster while controlling for confounders, including age, gender, house structure, and other socioeconomic status indicators.

For secondary outcomes, the PCR malaria prevalence of all the age groups, the microscopic malaria prevalence, the proportion of participants using the bed net, and the malaria knowledge of the adult participants will be evaluated similarly.

### Interim analyses {21b}

Since no interim analysis or stopping guidelines have been planned. From the aspect of the benefit of the population, a stepped wedge design will be followed after this trial in the case that we confirm the net positive benefits of the intervention.

### Methods for additional analyses (e.g., subgroup analyses) {20b}

As malaria infection risk differs depending on socioeconomic status [16], we are also interested in whether the intervention effects could differ across socioeconomic status. Using socioeconomic variables collected in the baseline survey, such as the wealth index of households and education level of household heads, we will do subgroup analyses. For the additional analyses, we also use the regression model for two-stage randomized experiments [11].

### Methods in analysis to handle protocol non-adherence and any statistical methods to handle missing data {20c}

The extent and patterns of missing data will be assessed once all data collection has been completed. If necessary, multiple imputation methods will be used to handle missing data.

### Plans to give access to the full protocol, participant-level data, and statistical code {31c}

This manuscript is the full protocol. The corresponding author will make the de-identified datasets or any future statistical code available upon reasonable request.

## Oversight and monitoring

### Composition of the coordinating center and trial steering committee {5d}

The sampling team, composed of CHVs and laboratory technicians, set up a day-to-day communication group and exchanged their experiences. A local management team of study investigators from Kenya and Japan also joined this, leading and advising the activities and monitoring the sample and data integrity. A monthly meeting will be held by the steering committee composed of all key researchers from Kenya and Japan, including the principal investigator (PI) and co-PI, which aim to monitor the progress of the trial.

### Composition of the data monitoring committee, its role and reporting structure {21a}

Because this intervention is considered to be of a low-risk nature, this study does not have a data monitoring committee. For additional credibility regarding study quality, the researchers will consult a third statistician if necessary.

### Adverse event reporting and harms {22}

Neither the financial incentive nor the malaria education is known to pose significant health or safety risks. Nonetheless, all unanticipated problems will be reported to the research team and Homa Bay County Ministry of Health (MOH) through CHVs. Medical officers from Homa Bay County will assess the relatedness of the reported events to the study and report to the research team, including the PI. In the event of a study-related serious adverse event, the study team will convene a meeting immediately with the MOH and Homa Bay County Teaching and Referral Hospital representatives to review the case and take necessary action.

### Frequency and plans for auditing trial conduct {23}

After participant recruitment, enrollment, and implementation of the intervention are completed, the research team will have a meeting to review the protocols for outcome evaluation. A monthly meeting will be held during the follow-up period to ensure that all surveys and investigations are conducted according to the study protocol. The study is required to submit annual reports and renewal to ethical review boards of Otaru University of Commerce, Japan, and Mount Kenya University, Kenya.

### Plans for communicating important protocol amendments to relevant parties (e.g., trial participants, ethical committees) {25}

Decisions on important trial amendments must be made through a formal procedure and will be approved by institutional review boards (IRB) at Mount Kenya University and Otaru University of Commerce. The protocol in the clinical trials registry will also be updated accordingly.

### Dissemination plans {31a}

The results will be shared with the Homa Bay County Government and Kenya National Malaria Control Program and discussed for the possibility of expanding the program. Also, the results will be disseminated through publications and conferences to help the development of novel malaria control strategies in other malaria-endemic countries. The feedback from the research participants will also help shape the future improvement of the intervention and acceptance by the communities.

## Discussion

Since around 2000, the United Nations/Millennium Development Goals (MDGs) and the establishment of the Global Fund have increased funding for global malaria control, and there have been significant improvements in the supply side of healthcare services for malaria control, including the distribution of long-lasting insecticidal nets (LLINs), implementation of indoor residual spraying (IRS), rapid diagnostic test (RDT), and artemisinin-based combination therapy (ACT) in community health facilities. In fact, the number of malaria cases and deaths worldwide has decreased significantly to the point where malaria elimination by 2030 was targeted as one goal in the 2015 United Nations Sustainable Development Goals (SDGs). However, while there has been a marked contraction of malaria prevalence in Southeast Asian countries, tropical Africa continues to experience high malaria prevalence. Indeed, Suba South Sub-county, Homabay County, Kenya, located in the Lake Victoria basin, has shown high malaria prevalence despite the intensive malaria control efforts by national and international aid agencies. One possible major cause of this residual transmission is the low motivation of the residents for malaria prevention and early treatment. For example, in the target areas, residents have been observed using mosquito nets distributed free of charge for purposes other than malaria control.

In this study, we aim to develop a policy tool that alters residents’ attitudes and behaviors for malaria elimination. Our proposed plan is a financial incentive intervention for residents in malaria-prone areas complemented with malaria education. The intervention includes two components. The first component is the introduction of a financial incentive scheme tied to negative RDT results for malaria infection among the target population. This is an attempt to promote behavioral changes in the population by providing monetary incentives. The project has two different forms of incentive schemes. One is a conditional cash transfer (CCT) that offers a small reward (200 Ksh) for non-infected subjects during the follow-up survey, and the other is a lottery incentive scheme (LIS) that gives a lottery with a 10% chance of winning a large reward (2000 Ksh) instead of the small reward. The CCT is an incentive scheme widely used in educational or medical aid interventions to promote behavioral changes in a target population. On the other hand, The LIS is a relatively new scheme with few applications and a variant of the CCT that uses findings from the behavioral sciences. Specifically, the LIS scheme utilizes the finding on subjective probability in behavioral science that people tend to overestimate the probability of events with a small probability of occurrence [17–19]. Suppose such a tendency exists among many people. In that case, the LIS may incentivize people more than the CCT. Moreover, if the LIS works as an incentive scheme, it also has a practical advantage against the CCT. Since the reward is paid with probability to a pool of candidates who meet the payment conditions in the LIS, the number of people receiving rewards is much less in the LIS than in the CCT, thus reducing the operational cost [20].

The second component is malaria education using animated tablet-based malaria educational material (EDU) developed by the research team. It complements the incentive scheme by providing the appropriate knowledge to the residents for malaria elimination. The educational content includes the medical knowledge necessary for malaria control, such as disease characteristics, transmission mechanisms, and prevention methods. Also, it explains asymptomatic and sub-microscopic malaria infection, its high prevalence in the region, and the risk of transmission from asymptomatic infected individuals, which have recently attracted attention as obstacles to malaria control among experts, although the local population does not know [21].

Furthermore, it includes the economic losses caused by malaria disease and the benefits of prevention and early treatment. The content introduces the direct and indirect costs of treatment for malaria disease, lost income due to absence from work, the decline in children’s academic performance caused by school absence, and the possibility of loss of future opportunities, based on the findings from the existing economics literature.

It is worth discussing the rationale for providing financial incentives to residents for malaria prevention. Firstly, in infectious diseases such as malaria, individuals’ actions for prevention and early treatment benefit their neighbors because such actions lower the infection risk of the neighbors. People often do not consider such spillover effects when they decide their effort level for malaria control. Thus, voluntary efforts of individuals to prevent infection tend to be lower than the socially optimal level [8–10]. Financial incentives may enhance individuals’ efforts for malaria prevention toward the socially optimal level.

Secondly, currently, massive private and public resources have been used for malaria control and treatment. The financial incentives may be able to save such resources for malaria control by lowering malaria risks in the region. According to our census data in 2021 in the region, for example, 17.2% of working adults (above 15 years old) had at least one malaria episode during the last 12 months, where we counted only cases diagnosed by malaria tests, spent 120 Ksh for transport to health facility and 480 Ksh for treatment on average, and took 7.7 days for the recovery. Working adults get 260 Ksh daily and work 5 days a week on average. A simple calculation tells us that one malaria episode of a working adult costs 2030 Ksh (120 for transport, 480 for treatment, and 1430 for foregone earnings) on average. If the cost of the public resource for their treatment is counted and also if the cost of family members and neighbors who get malaria infection from them is counted, the total cost of one malaria episode will be much more than our calculation. Thus, we believe that social gain from reducing malaria risk could be significant, and hence, it is valuable to consider a policy tool such as the financial incentive intervention to alter the residents’ behaviors for malaria prevention.

The cost calculation also gives us a rough idea of at which level to set the reward in our financial incentive intervention. Our intervention set the reward amount at 200 Shillings for the CCT and 2000 Shillings with a winning probability of 10% for the LIS. The expected amount of reward is the same between the schemes for comparison purposes and also similar to the expected cost that an individual adult has to incur for malaria treatment for a period of 6 months (which is the duration from the intervention to the follow-up survey). As mentioned earlier, on average, the cost of one malaria episode in an adult was 2030 Shillings. The average number of malaria infections per adult would be 0.094 for 6 months, given the probability of having at least one malaria episode for 12 months (17.2%) under the assumption of the Poisson process on the number of individual malaria infections. This simple calculation gives us the expected private cost of approximately 190 Shillings for malaria treatment for 6 months.

This study has several limitations. First, the financial incentive schemes are designed so that the monetary rewards are tied to the negativity of RDT in the follow-up rather than that of the PCR despite the low sensitivity of RDT, especially for low parasitemia cases. This is due to a practical reason. It takes only 15 min to obtain the RDT results, and hence, both participants and survey teams can observe the RDT results together and the reward status for the eligible household members on the spot. On the other hand, the PCR results are obtained only in a well-equipped laboratory and need several days, including the transportation and sorting of blood samples. The verifiability of the reward condition on the spot is important for the participants to trust in the incentive scheme since if the participants do not trust it, they will not respond to it. However, we should cautiously consider that the rewards would be given to those with asymptomatic and sub-microscopic infection if the RDT fails to detect such infection, which happens more often than the PCR. Ideally, we should not give rewards for such cases since sub-microscopic infection is the one we also want the participants to avoid. We will continue monitoring the participants’ behaviors after the follow-up survey.

Second, there are some neighboring clusters with almost a negligible buffer, and hence, there is a possibility of inter-cluster influence due to the close proximity of physical and social distance between participants in neighboring clusters. This possibility may violate the stable unit treatment value assumption (SUTVA). Thus, we may need to consider a method to estimate the treatment effect under the possibility of the violation of the SUTVA by following a recent study [22].

Third, the household-level randomization for the treatment assignment will be done by asking the household representative to draw a card with a sealed number during the household visit in the intervention clusters, and the treatment status will be determined by the number on the card and known by the participants. This may cause non-random attrition in the follow-up survey due to the reluctance of those who fail to get the treatment to participate in the follow-up survey. We may need to handle the sample attrition carefully because it can be associated with the treatment status within the intervention clusters.

This study will be the first trial to evaluate the financial incentive schemes on the infection incidence by active case detection and the malaria preventive behaviors of the residents in a malaria-prone area. Results from this study will inform decision-makers who seek an effective policy tool for malaria elimination.

## Trial status

Recruitment started on January 7, 2022, and the final subject enrolment was completed on March 8, 2022. The intervention started on June 18, 2022, and was completed on July 29, 2022. The follow-up survey started on December 19, 2022, and completed on February 10, 2023. The focus group discussion and questionnaire survey for the perception, as a final data collection, is planned for December 2023 (Table 3). The current protocol is version 1.0 of December 1, 2022. The study was initially intended to be a stepped wedge trial. However, the authors agreed that the study should be published as a stand-alone cluster-randomized controlled trial; thus, submission of this protocol was delayed.

**Table 3:** Trial status and plan

Action	Date
The date of the first enrolment (baseline)	January 7, 2022
Finalize the enrolment (baseline)	March 8, 2022
Start the implementation of the intervention	June 18, 2022
Finalize all the implementation of the intervention	July 29, 2022
Start the follow-up survey	December 19, 2022
Complete the follow-up survey	February 10, 2023
Qualitative data collection (Completion of the data collection) (expected)	December 2023

## Data Availability

The study regimes, consent forms, assent forms, and study-related materials are accessible from the corresponding author. The final trial dataset will be available to all investigators. The corresponding author will make the de-identified datasets available upon reasonable request.

## Appendix

### The English translation of the intervention script

The following script is used for all the target households:

*We would like to ask all of you who have been cooperating in this research project to continue to join it. As you all know, Homabay County has a very high rate of malaria prevalence. The most effective prevention method is the proper use of insecticide-treated mosquito nets. However, it is not being thoroughly implemented. If we implement appropriate preventive actions, including the use of mosquito nets, and early diagnosis and treatment in health facilities when the infection is suspected, we should be able to contain malaria transmission in the region.*

The following script is used for the households in treatment clusters (CCT and LIS):

*In order to encourage such appropriate preventive actions, this research project will experiment with an educational tool to increase knowledge about malaria and a reward scheme for your efforts to avoid contracting malaria. The reward scheme is to reward those who are confirmed to be malaria-negative at the next survey scheduled in 6 months when the RDT test is conducted. All persons listed under this household in this survey are eligible.*

*Please note that this is an experimental trial with a limited budget; not all households can be eligible for the reward this time. This time, 50% of the households who cooperate with the survey will be eligible for the reward scheme.*

*Whether or not a household is eligible for the reward system will be determined by a scratch card. We will ask a representative of the household to draw a card, and if a card with an even number is picked, the household will be eligible for the reward scheme.*

*If this experiment is successful, we will consider continuing the reward scheme and implementing policies to target more households.*

*If you agree, you will be asked to pick a scratch card. A scratch card has a number from 1 to 10 printed under the seal. You and your family members are eligible for the reward scheme if you draw a card with an even number, i.e., 2, 4, 6, 8, or 10.*

*Please choose who will draw a scratch card?*

[Enumerator Instruction] Ask a respondent to draw a card from the envelope with 10 cards. Once s/he draws a card, remove the seal by a coin in front of them and share the result with them.

The following script is used for the eligible households in CCT clusters:

*Congratulations! You drew a card with an even number. Your household members are eligible for the reward scheme. We will explain it in detail.*

*The reward will be 200 shillings per person. This means that if the RDT test at the time of your next survey scheduled in 6 months is confirmed negative, we will pay you 200 shillings per person in MPESA. This will apply to all the members who are listed under this household in this survey. For example, if you have a family of five, and all five are confirmed negative at the next survey, we will reward you with 1,000 shillings to your registered MPESA account. We encourage you to take preventive measures against malaria properly. Even if you have a symptom suspected of malaria, e.g., fever, you can immediately visit a health center and get an early diagnosis and treatment. Then, you will be eligible for the reward if you remain negative at the next survey time. Please earn the reward by complying with prevention and early diagnosis and treatment. If you test positive, an anti-malaria treatment will be provided according to the Government guidelines as before instead of a reward.*

*Do you understand it?*

[Enumerator Instruction] If the respondent says NO, explain it again.

The following script is used for the eligible households in LIS clusters:

*Congratulations! You drew an even number. Your household members are eligible for the reward scheme. We will explain it in detail.*

*The reward will be a lottery with the possibility of winning 2000 shillings. In other words, those who have a negative RDT test on the next survey scheduled in 6 months will get a lottery for a chance to win 2,000 shillings. If you win the lottery, we will pay you 2000 shillings in MPESA. This will apply to all the members who are listed under this household in this survey. The lottery is a scratch card similar to the one used just before. The scratch cards have numbers from 1 to 10 printed on them. If you get a specific number, 10, you win. Therefore, the chance of winning is 1 out of 10. For example, if you have a family of five people cooperating with us, we will give you five lotteries for the whole family if all five people are confirmed negative in the next survey. If one of them is a winner, we will pay you 2,000 shillings, and if two of them are winners, we will pay you 4,000 shillings to your registered MPESA account. We encourage you to take preventive measures against malaria properly. Even if you have a symptom suspected of malaria, e.g., fever, you can immediately visit a health center and get an early diagnosis and treatment. Then, you will be eligible for the reward if you remain negative at the next survey time. Please earn the reward by complying with prevention and early diagnosis and treatment. If you test positive, an anti-malaria treatment will be provided according to the Government guidelines as before instead of a reward.*

*Do you understand it?*

[Enumerator Instruction] If the respondent says NO, explain it again.

## Abbreviations

LLIN: Long-lasting insecticide-treated net
PBO: Piperonyl butoxide
ITN: Insecticide-treated net
RDT: Rapid diagnostic test
ACT: Artemisinin combination therapy
CHV: Community health volunteer
CCT: Conditional cash transfer
LIS: Lottery incentive scheme
EDU: Malaria education using the original animated tablet-based material
